# Tocilizumab improves 28-day survival in hospitalized patients with severe COVID-19: an open label, prospective study

**DOI:** 10.1186/s12931-021-01914-6

**Published:** 2021-12-22

**Authors:** Theodoros Karampitsakos, Elli Malakounidou, Ourania Papaioannou, Vasilina Dimakopoulou, Eirini Zarkadi, Matthaios Katsaras, Panagiota Tsiri, Georgios Tsirikos, Vasiliki Georgiopoulou, Ioanna Oikonomou, Christos Davoulos, Dimitrios Velissaris, Fotios Sampsonas, Markos Marangos, Karolina Akinosoglou, Argyris Tzouvelekis

**Affiliations:** 1 Department of Respiratory Medicine, University Hospital of Patras, Pátrai, Greece; 2 Department of Internal Medicine, University Hospital of Patras, Pátrai, Greece

**Keywords:** COVID-19, Tocilizumab, Mortality, PaO_2_/FiO_2_, Outcomes

## Abstract

**Background:**

Data on the safety and efficacy profile of tocilizumab in patients with severe COVID-19 needs to be enriched.

**Methods:**

In this open label, prospective study, we evaluated clinical outcomes in consecutive patients with COVID-19 and PaO_2_/FiO_2_ < 200 receiving tocilizumab plus usual care versus usual care alone. Tocilizumab was administered at the time point that PaO_2_/FiO_2_ < 200 was observed. The primary outcome was 28-day mortality. Secondary outcomes included time to discharge, change in PaO_2_/FiO2 at day 5 and change in WHO progression scale at day 10.

**Findings:**

Overall, 114 patients were included in the analysis (tocilizumab plus usual care: 56, usual care: 58). Allocation to usual care was associated with significant increase in 28-day mortality compared to tocilizumab plus usual care [Cox proportional-hazards model: HR: 3.34, (95% CI: 1.21–9.30), (p = 0.02)]. There was not a statistically significant difference with regards to hospital discharge over the 28 day period for patients receiving tocilizumab compared to usual care [11.0 days (95% CI: 9.0 to 16.0) vs 14.0 days (95% CI: 10.0–24.0), HR: 1.32 (95% CI: 0.84–2.08), p = 0.21]. ΔPaO_2_/FiO_2_ at day 5 was significantly higher in the tocilizumab group compared to the usual care group [42.0 (95% CI: 23.0–84.7) vs 15.8 (95% CI: − 19.4–50.3), p = 0.03]. ΔWHO scale at day 10 was significantly lower in the tocilizumab group compared to the usual care group (-0.5 ± 2.1 vs 0.6 ± 2.6, p = 0.005).

**Conclusion:**

Administration of tocilizumab, at the time point that PaO_2_/FiO_2_ < 200 was observed, improved survival and other clinical outcomes in hospitalized patients with severe COVID-19 irrespective of systemic inflammatory markers levels.

**Supplementary Information:**

The online version contains supplementary material available at 10.1186/s12931-021-01914-6.

## Introduction

The spread of 2019 coronavirus disease (COVID-19) and the associated acute respiratory distress syndrome (ARDS) are responsible for the worst public health crisis of the latest century [1]. Despite major advances in the management of COVID-19, a considerable proportion of infected individuals experiences critical illness with hypoxic respiratory failure requiring prolonged ventilatory support [2–5]. Hypoxic respiratory failure in patients with COVID-19 has been associated with release of pro-inflammatory cytokines including interleukin (IL)-6 [6]. IL-6 has been implicated in endothelial and vascular dysfunction by inducing acute phase reactants such as C reactive protein (CRP), hepcidin and fibrinogen from hepatocytes, as well as through induction of T cell differentiation and antibody production [7–9]. Higher levels of IL-6 have been associated with COVID-19 severity [10].

A growing body of evidence support the concept of an excessive host inflammatory response leading to critical illness and increased mortality. In line with this, low doses of corticosteroids have been the only, so far, therapeutic approach that consistently improves survival across multiple studies [2, 11, 12]. Tocilizumab is a recombinant, humanized anti-IL-6 receptor monoclonal antibody initially launched as an intravenous treatment for rheumatoid arthritis, giant cell arteritis and chimeric antigen receptor T-cell-induced severe cytokine release syndrome [13–15]. The largest to date study, denominated RECOVERY trial, showed survival benefit for patients with COVID-19 receiving tocilizumab versus usual care alone [16]. However, other studies yielded rather contradictory results [16–24]. Inclusion criteria might be major contributors of this discrepancy. In particular, administration of tocilizumab in all hospitalized patients irrespective of disease severity may have diluted any potential therapeutic effects in specific subpopulations of patients, including critically-ill patients. In addition, using arbitrary values of non-specific inflammatory markers such as CRP to tailor therapeutic approaches and prioritize patients for treatment has yielded poor results, so far. Timing from clinical presentation to treatment varied among trials and thus there was an unmet need to address whether timing of administration influences the efficacy of tocilizumab.

Our aim was to prospectively investigate the safety and efficacy profile of tocilizumab administration at the time point that PaO2/FiO2 < 200 was observed in hospitalized patients with COVID-19 irrespective of CRP and other markers of systemic inflammation.

## Methods

### Trial design and oversight

We conducted an open-label, prospective study enrolling consecutive patients with positive PCR for SARS-CoV-2 admitted to our hospital between 15/2/2021 and 21/4/2021. Trial sites were two separate COVID-19 departments of University Hospital of Patras, Greece. Patients were assigned to one of the two departments on 1:1 ratio following examination in the emergency unit. Of note, clinicians of these two departments were experienced in management of COVID-19, shared common algorithms and had been selected from the Department of Respiratory Medicine or the Department of Internal Medicine. Patients aged 18 years or older that presented with PaO_2_/FiO_2_ < 200 at any time during their hospitalization were included in the analysis irrespective of values in inflammatory markers, such as CRP and ferritin. Exclusion criteria were: age < 18 years, pregnancy and application of mechanical ventilation prior patients’ transfer to our Hospital. Each patient or the patient’s legally authorized representative provided written or witnessed oral informed consent. The trial was conducted in accordance with the International Conference on Harmonisation E6 guidelines for Good Clinical Practice, the Declaration of Helsinki and the local regulations. Our study was approved by our Institutional Review Board and the Local Ethics Committee (Protocol Number: 9273/06-04-21).

**Fig. 1:**
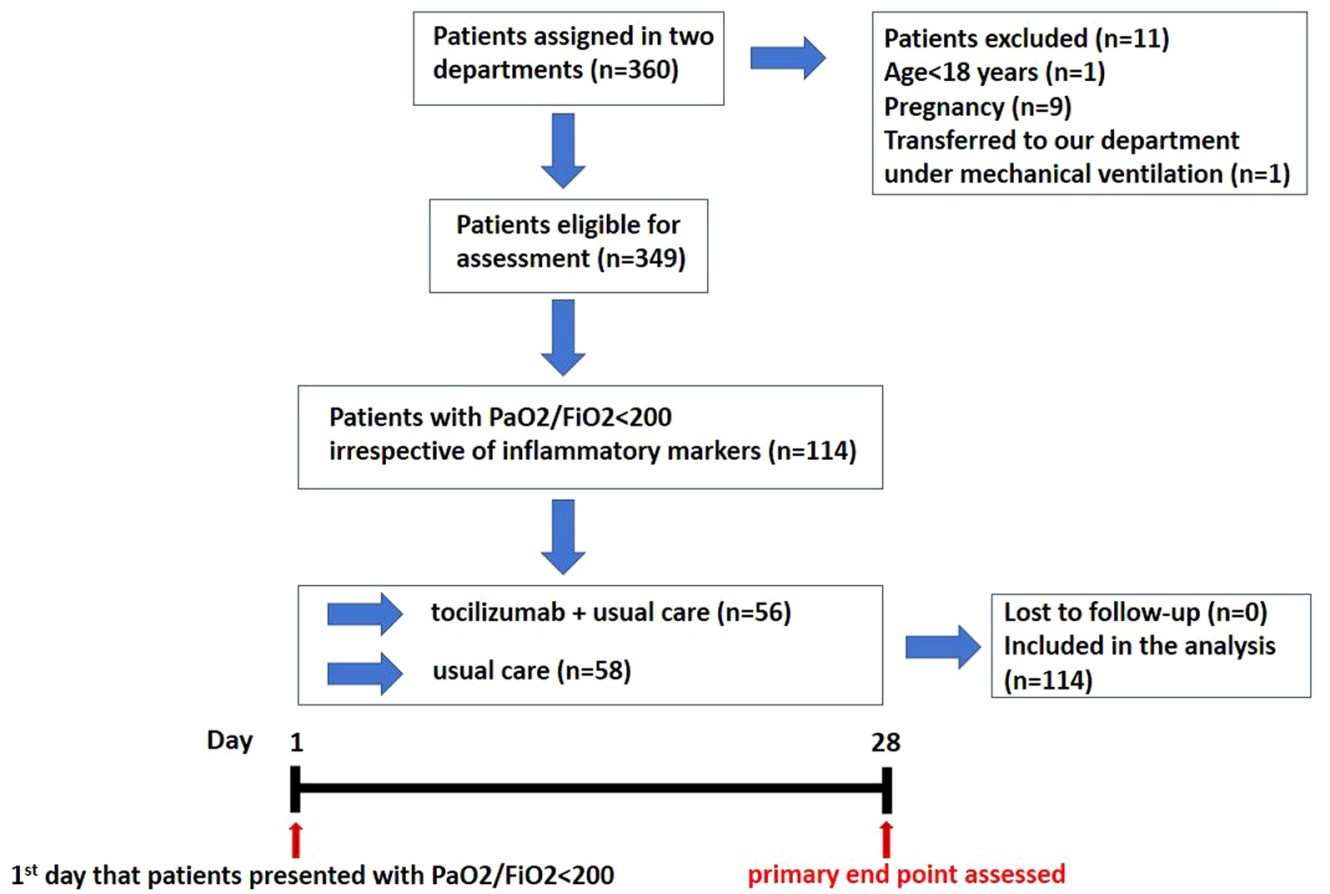
Schematic representation of the study design

Day 1 was considered the first day when a patient reached a PaO_2_/FiO_2_ < 200. Depending on the department assigned, patients with PaO_2_/FiO_2_ < 200 received usual care alone (department 1) or tocilizumab plus usual care (department 2). Tocilizumab was administered intravenously (IV) at 5 mg/kg the first day that patients presented with PaO_2_/FiO_2_ < 200 (day 1). All patients included in the analysis received dexamethasone at a dose of 6 mg/day and the dose did not change in any patient. Usual care also included remdesivir (in all patients except cases that was contra-indicated), antibiotic compounds, vasopressor support and anticoagulants that were provided at the discretion of the clinicians. Usual care did not include other compounds such as baricitinib, convalescent plasma, nintedanib and pirfenidone. The study design is summarized in Fig. 1.

### Outcome measures

Our primary end point was mortality by day 28. Our secondary outcomes were time to discharge and disease progression in multiple time points, as indicated by change in PaO_2_/FiO_2_ [ΔPaO_2_/FiO_2_ (day 5–day 1)] at day 5 and change in WHO clinical progression scale at day 10[ΔWHO scale (day 10–day 1)]. Towards this direction, we recorded PaO_2_/FiO_2_ of each patient, as well as demographics, smoking status, comorbidities, complete blood count, biochemical parameters, WHO clinical progression scale, time to discharge and time to event (mortality). WHO clinical progression scale provided a measure of illness severity across a range from 0 (not infected) to 10 (dead) with data elements that are rapidly obtainable from clinical records [25]. The incidence and severity of adverse events were also evaluated. These events were determined according to the National Cancer Institute Common Terminology Criteria for Adverse Events, version 5.0.

### Statistical analysis

Continuous data were denoted as mean ± standard deviation (SD) or medians with 95% Confidence Interval (95% CI) following Kolmogorov–Smirnov test for normality. The primary outcome was estimated with the Kaplan–Meier method and cumulative incidence curves were compared between the two groups. The stratified Cox proportional-hazards model was used to estimate the hazard ratio and 95% CI. Time-to-discharge secondary outcome was compared between the two groups with the use of the Kaplan–Meier approach and cumulative incidence curves were compared between the two groups. We assigned the ‘worst outcome’ for individuals who died before day 28 and thus these patients were right-censored at the longest hospital stay [26]. Mann Whitney or t-test were used for the investigation of differences in ΔPaO_2_/FiO_2_ and ΔWHO scale between the two groups based on the absence or presence of normality. ΔPaO_2_/FiO_2_ at day 5 was measured only for patients receiving the same method of oxygen therapy [Conventional oxygen therapy, High-flow nasal cannula, Continuous-Positive Airway Pressure (C-PAP)] during the first 5 days in order to avoid erroneous interpretations from the effect of positive end expiratory pressure (PEEP).

In an effort to validate the role of ΔPaO2/FiO2 at day 5 as a secondary outcome, we used the Kaplan–Meier method to assess the prognostic performance of ΔPaO_2_/FiO_2_ at day 5 in the overall population, irrespective of treatment. In particular, patients were split by the median value of ΔPaO_2_/FiO_2_ at day 5 irrespective of treatment. Subsequently, the Kaplan–Meier method estimated survival probability in the high versus low ΔPaO_2_/FiO_2_ group. p-values < 0.05 were considered statistically significant. Results were illustrated in tables and figures.

## Results

### Patients

Three hundred sixty patients (n = 360) patients were assigned in both departments during the study period. Eleven patients (n = 11) met the exclusion criteria. Three hundred forty-nine patients (n = 349) were eligible for assessment. Among patients eligible for assessment, 114 (32.7%) presented with PaO_2_/FiO_2_ < 200 during hospitalization and were included in the analysis. Fifty-six (n = 56) patients were assigned to receive tocilizumab plus usual care and 58 were assigned to receive usual care (Fig. 1). We had no missing data (0%) for patients included in the analysis. PCR analysis revealed that all cases included in our study were infected by alpha variant, which was the predominant variant in Greece by the time of the study. The majority of patients were men in both groups [tocilizumab group: n = 33 (58.9%), usual care group: n = 36 (62.1%)]. Median age was 66.0 years (95% CI: 60.0–70.0) and 66.5 years (95% CI: 62.1–72.0) for tocilizumab and usual care group, respectively. Baseline demographic and disease characteristics were generally balanced between the two groups (Table 1).

**Table 1:** Characteristics of the patients at baseline

Characteristics	Tocilizumab group (N, %)	Usual care group (N, %)	p value
Number of patients Age (median, %95 CI) Male sex/Female sex Current/ Ex-smokers/Never smokers Arterial hypertension Dyslipidemia Diabetes mellitus Depression Obesity Hypothyroidism Chronic heart disease Cancer WHO clinical progression scale day1(mean ± SD) PaO_2_/FiO_2_ day 1 (median, 95% CI) C-reactive protein (mg/dL, median, 95% CI) D-dimer (μg/ml, median,95% CI) Ferritin (ng/ml, median, 95% CI) White blood cells (/μL,median,95% CI) Polymorphonuclear leukocytes (/μL, median, 95% CI) Lymphocytes (/μL, median, 95% CI) RDW (%, median, 95% CI)	56 66.0 (60.0 to 70.0) 33 (58.9%)/23 (41.1%) 5 (8.9%)/18 (32.1%)/33 (58.9%) 29 (51.8%) 17 (30.3%) 13 (23.2%) 11 (19.6%) 9 (16.1%) 8 (14.3%) 6 (10.7%) 4 (7.1%) 5.3 ± 0.5 154.5 (130.0 to 165.0) 7.5 (4.7 to 9.6) 0.82 (0.61 to 1.18) 765.0 (545.5 to 1191.9) 6190.0 (5467.9 to 6882.2) 5120.0 (4319.9 to 5688.0) 735.0 (654.0 to 818.0) 14.2 (13.5 to 14.4)	58 66.5 (62.1 to 72.0) 36 (62.1%)/22 (37.9%) 10 (17.2%)/ 13 (22.4%)/35 (60.3%) 25 (43.1%) 7 (12.1%) 14 (24.1%) 6 (10.3%) 6 (10.3%) 6 (10.3%) 11 (18.9%) 6 (10.3%) 5.1 ± 0.5 157.0 (131.9 to 162.9) 8.5 (6.5 to 11.5) 0.84 (0.65 to 1.19) 644.5 (493.3 to 837.1) 5915.0 (5400.0 to 7657.2) 4565.0 (3936.7 to 5857.0) 815.0 (692.7 to 920.0) 14.0 (13.4 to 15.1)	NA 0.51 0.72 0.19/0.25/0.88 0.35 0.02 0.91 0.16 0.36 0.52 0.22 0.55 0.75 0.69 0.09 0.80 0.43 0.84 0.75 0.13 0.21

*CI* Confidence Interval, *COPD* Chronic Obstructive Pulmonary Disease, *RDW* Red cell distribution width, *SD* Standard Deviation

At day 1, 37 patients (66.1%) in the tocilizumab group received conventional oxygen therapy and 19 patients (33.9%) received High-flow nasal cannula or C-PAP. Accordingly, 48 (82.8%) and 10 (17.2%) patients in the usual care group received conventional oxygen therapy and High-flow nasal cannula or C-PAP at day 1, respectively. At day 5, 26 patients (46.4%) in the tocilizumab group received conventional oxygen therapy, 23 (41.1%) patients received High-flow nasal cannula or C-PAP, 5 patients (8.9%) had been discharged alive and 2 patients (3.6%) were mechanically ventilated or dead. In the usual care group, 32 patients (55.2%) received conventional oxygen therapy, 17 patients (29.3%) received High-flow nasal cannula or C-PAP, 3 patients (5.2%) had been discharged alive and 6 patients (10.3%) were mechanically ventilated or dead.

### Primary efficacy outcome

**Fig. 2:**
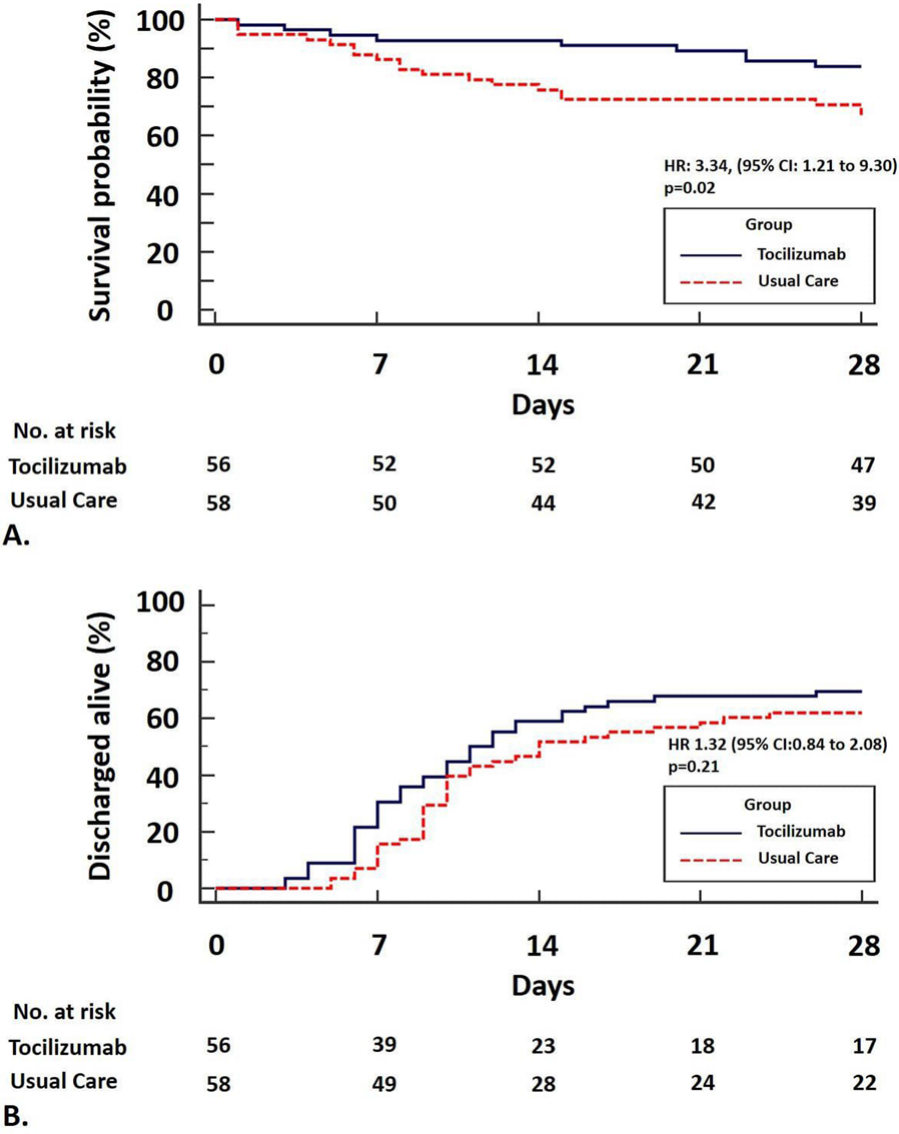
Allocation to usual care was associated with significant increase in 28-day mortality compared to tocilizumab plus usual care [Cox proportional-hazards model: HR: 3.34, (95% CI: 1.21–9.30), (p = 0.02)], (**A**). There was not a statistically significant difference with regards to hospital discharge over the 28-day period for patients receiving tocilizumab compared to usual care [11.0 days (95% CI: 9.0–16.0) vs 14.0 days (95% CI: 10.0–24.0), HR: 1.32 (95% CI: 0.84–2.08), p = 0.21], (**B**)

**Fig. 3:**
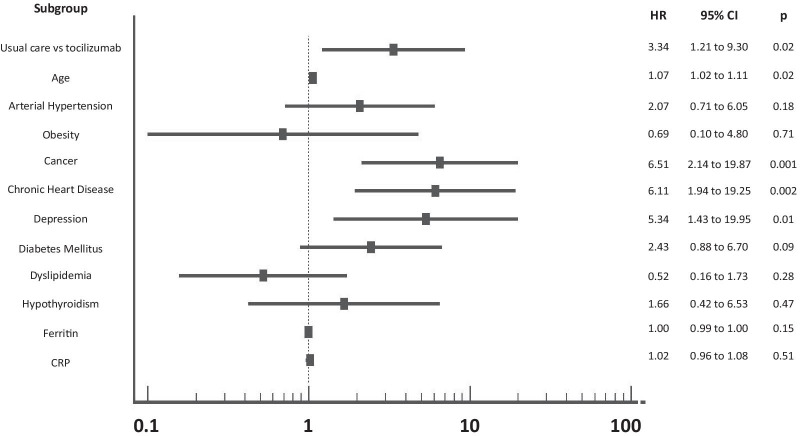
Forest plot of the primary endpoint (mortality) in prespecified subgroups. Estimates are based on a Cox proportional-hazards model

The cumulative percentage of patients who reached the endpoint of mortality by day 28 was significantly lower in the tocilizumab group (16.1%) than in the usual care group (32.8%) [Cox proportional-hazards model for survival probability: HR: 3.34, (95% CI: 1.21 to 9.30), (p = 0.02)]. Results are shown in Table 2, Fig. 2A and Fig. 3.

**Table 2:** Primary and secondary efficacy outcomes by day 28

Efficacy outcomes	Tocilizumab group (N = 56)	Usual care group (N = 58)	p value
Primary outcome			
Mortality (patients: N, %)	9 (16.1%)	19 (32.8%)	**0.03**
Secondary outcomes			
Time to discharge (days)/patients discharged alive by day 28 (N, %)	11.0 (95% CI: 9.0 to 16.0)/39 (69.6%)	14.0 (95% CI:10.0–24.0)/36 (62.1%)	0.21
ΔPaO_2_/FiO_2_ ( Day 5–Day1)	42.0 (23.0–84.7)	15.8 (− 19.4–50.3)	**0.03**
ΔWHO scale (Day 10–Day 1)	− 0.5 ± 2.1	0.6 ± 2.6	**0.005**

Bold numbers are statistically significant

### Secondary efficacy outcomes

The median time to hospital discharge over the 28-day period was 11.0 days (95% CI: 9.0 to 16.0) in the tocilizumab group and 14.0 days (95% CI: 10.0 to 24.0) in the usual care group [HR: 1.32 (95% CI: 0.84 to 2.08), p = 0.21] (Fig. 2B). Thirty nine (n = 39, 69.6%) and 36 patients (62.1%) had been discharged alive by day 28 in the tocilizumab and usual care group, respectively (Table 2). Eleven (n = 11, 19.6%) and 14 patients (24.1%) received mechanical ventilation by day 28 in the tocilizumab and usual care group, respectively.

ΔWHO scale at day 10 was significantly lower in the tocilizumab group compared to the usual care group (-0.5 ± 2.1 vs 0.6 ± 2.6, p = 0.005), (Additional file 1: Fig. S1A). ΔPaO_2_/FiO_2_ at day 5 was measured only for patients receiving the same method of oxygen therapy during the first 5 days (overall population = 83, tocilizumab group = 41, usual care croup = 42). ΔPaO_2_/FiO_2_ at day 5 was significantly higher in the tocilizumab group compared to the usual care group [42.0 (95% CI: 23.0 to 84.7) vs 15.8 (95% CI: -19.4 to 50.3), p = 0.03], (Additional file 1: Fig. SB). Multiple regression analysis showed that besides tocilizumab use, baseline ferritin levels were also associated with ΔPaO2/FiO2 at day 5 (p = 0.02).

In the overall population, median ΔPaO_2_/FiO_2_ at day 5 was 35 (95% CI: 16.9 to 60.0). Kaplan–Meier analysis in the overall population irrespective of treatment arm demonstrated higher all-cause mortality in patients with high (≥ 35) vs. low ΔPaO_2_/FiO_2_ at day 5 (< 35), [HR 3.70 (95% CI: 1.24 to 11.00), (p = 0.03)], (Additional file 1: Fig. S2).

### Adverse events

Adverse events by day 28 were comparable between the two groups. In particular, 3 patients (5.4%) presented with lobar consolidation, while cardiac event and bleeding occurred in 1 patient (1.8%) in the tocilizumab group. Two patients (3.4%) presented with lobar consolidation, while cardiac event and bleeding occurred in 1 patient (1.7%) in the usual care group (Table 3).

**Table 3:** Adverse events by day 28

Adverse events	Tocilizumab group (N = 56)	Usual care group (N = 58)
Lobar consolidation Cardiac Event Bleeding Septic shock	3 (5.4%) 1 (1.8%) 1 (1.8%) 0 (0%)	2 (3.4%) 1 (1.7%) 1 (1.7%) 0 (0%)

## Discussion

To the best of our knowledge, this is the first study aiming to address the question whether administration of tocilizumab at the time point that PaO_2_/FiO_2_ < 200 was observed influences its efficacy. We included a homogenized population of patients with severe COVID-19 and administered tocilizumab at the time of clinical and physiological deterioration, as assessed by PaO_2_/FiO_2_ < 200 irrespective of levels of non-specific inflammatory markers including CRP and ferritin. Treatment with tocilizumab improved survival and slowed down disease progression as indicated by changes in PaO_2_/FiO_2_ and WHO progression scale. Administration of tocilizumab at the time point of clinical and physiological deterioration deserves further investigation to tailor therapeutic approaches and identify those individuals who will most likely benefit from IL-6R antagonists.

Evidence from several relatively-small studies followed by meta-analyses yielded contradictory results on the safety and efficacy profile of tocilizumab in patients with COVID-19. In particular, a meta-analysis of the first eight randomized controlled trials (RCTs) (n = 2733) of tocilizumab failed to show a survival benefit [16–24]. On the contrary, the REMAP-CAP study, enrolling an overall of n = 803 critically ill COVID-19 patients demonstrated improved clinical outcomes compared to placebo [23]. The EMPACTA trial, recruiting patients with COVID-19 (n = 389) and blood oxygen saturation below 94%, showed reduced likelihood of progression to mechanical ventilation or death, but failed to reach significance on disease survival [20]. Most importantly, the largest so far tocilizumab study, the RECOVERY trial, encompassing an overall of 4116 patients, further enriched the beneficial effects of tocilizumab in all three primary end-points of hospitalization, survival and need for mechanical ventilation. Improved clinical outcomes were observed irrespective of the level of respiratory support [16].

The above contradictory results might be explained by several characteristics of the trials including heterogeneous enrollment criteria such as disease severity, timing of administration and concurrent use of corticosteroids [27]. Inclusion of patients with varying degree of disease severity might be linked with different degrees of inflammation with those being at the most advanced stage being these with the highest inflammatory status and thus expected to benefit more from anti-inflammatory interventions. On the other hand, patients at the most severe disease status with prolonged time since onset of symptoms may not benefit accordingly because inflammatory cascade may be too advanced to be reversible. The peak of the SARS-CoV-2 mediated inflammatory response often coincides with clinical deterioration [28]. Once the inflammatory cascade reaches a state of hyperactivation, it might be too late to intervene, and thus it has been suggested a time window within which IL-6R antagonists will be most beneficial. This window might correlate with the time around clinical deterioration, perhaps when organ dysfunction is more reversible [27]. Therefore, appropriate timing seems to be the most crucial contributor of treatment success. Based on the current literature, there was an unmet need to address the question whether timing of administration influences the efficacy of tocilizumab, as previous reports suggested that administration around the time of clinical deterioration might improve clinical outcomes; yet, none of the studies, so far, have been designed to confidently answer this question [27, 29].

Our study exhibited a number of important attributes by addressing these clinical sources of heterogeneity. In particular, tocilizumab was applied exactly at the time of clinical and physiological deterioration, as assessed by PaO_2_/FiO_2_ lower than 200, in a homogenized group of patients presenting with severe disease and concurrent use of corticosteroids. Cox proportional-hazards model showed that tocilizumab was efficacious irrespective of baseline condition; thus, some slight differences in baseline characteristics of the two groups did not affect outcomes. Our study results were in line with the REMAP-CAP study showing efficacy of tocilizumab across all CRP-subgroups [23]. Our data combined with others by no means support the notion that biologic enrichment of such studies will not be beneficial. Rather, we believe that biomarkers such as CRP or ferritin are neither sensitive, nor specific for treatment tailoring considering that their biologic association with lung inflammation is not linear and representative [30, 31]. On the other hand, physiological markers of local inflammation, such as PaO_2_/FiO_2_, might be more useful indicators of which patients will benefit from IL-6R antagonism [23, 32, 33]. Moreover, our approach might limit irrational use of tocilizumab and maximize cost-effectiveness. Otherwise, a dramatic upswing in prescribing will possibly challenge supply chains and health system budgets [34].

A novel finding of our study is the significant improvement of PaO_2_/FiO_2_ at day 5 for patients receiving tocilizumab plus usual care compared to usual care alone. Absence of serial data for PaO_2_/FiO_2_ was a limitation in previous RCTs of tocilizumab in COVID-19 [16]. We analyzed patients under constant method of oxygen therapy during the first 5 days of treatment initiation. In this way, we aimed to avoid misinterpretations arising from application of heterogeneous non-invasive respiratory therapies across the same patient. Despite the limitation that this analysis was performed in a proportion of the cohort, it seems that tocilizumab reduces the likelihood of physiological deterioration as reflected by step-up modalities of oxygen therapy. Moreover, we demonstrated the prognostic significance of change in PaO2/FiO2 at day 5 in patients with COVID-19. Finally, in the context of adverse events, consistent result across all trials to date, including our study, is that no increased incidence of serious adverse events have been reported for patients receiving tocilizumab [16–24, 35, 36].

Our study has some limitations that need to be treated cautiously. First, it was an open label trial. Although assessment of the primary outcome is objective, secondary outcomes such as score in WHO progression scale are operator-dependent. Limitations for the use of WHO progression scale as a secondary outcome include sensitivity to differences in local clinical practice, lack of proportionality between categories and lack of an established minimum clinically important difference. Second, our sample size was moderate; yet, adequate to detect significant differences in mortality. Third, patients were randomly assigned to one of the two departments based on the time of admission in the Emergency Unit; yet this was not a randomized controlled trial. Moreover, our study was not designed to assess radiological differences following tocilizumab treatment and thus this information is not included in our study. In addition, our study was designed to include patients for treatment with tocilizumab based on the PaO_2_/FiO_2_ ratio and not baseline IL-6 blood levels considering the limitations of treatment initiation based on arbitrary values of inflammatory markers including IL6 and CRP. Finally, the dosage we used (5 ml/kg) is lower than that of some other trials (8 ml/kg); nonetheless, this dose is within the range of the recommended tocilizumab dose (4–8 ml/kg). The concept that lower doses of tocilizumab might retain effectiveness and spare adverse events deserves further investigation.

## Conclusions

Collectively, the results of this open label, prospective study demonstrated that tocilizumab is an effective treatment for hospitalized patients with COVID-19 when administered at the time of clinical and physiological deterioration, as indicated by PaO_2_/FiO_2_ < 200. Improved clinical outcomes were observed across all treated patients irrespective of the magnitude of inflammation. Implementation of physiological markers of disease severity coupled with biological enrichment could help us identify the ideal time point and subgroup of patients with COVID-19 for optimal therapeutic and safety effects.

## Supplementary Information

**Additional file 1: Figure S1.** ΔWHO scale at day 10 was significantly lower in the tocilizumab group compared to the usual care group (− 0.5±2.1 vs 0.6±2.6, p=0.005), (**A**). ΔPaO_2_/FiO_2_ at day 5 was significantly higher in the tocilizumab group compared to the usual care group [42.0 (23.0–84.7) vs 15.8 (− 19.4–50.3), p=0.03], (**B**). **Figure S2.** Kaplan-Meier analysis in the overall population irrespective of treatment arm demonstrated higher all-cause mortality in patients with high (≥35) vs. low ΔPaO_2_/FiO_2_ at day 5 (< 5) [HR 3.70 (95% CI: 1.24–11.00), (p=0.03)].

## Data Availability

The datasets used and/or analysed during the current study are available from the corresponding author on reasonable request.
