# Supplementary material for: Tocilizumab improves 28-day survival in hospitalized patients with severe COVID-19: an open label, prospective study
Source: Respir Res. 2021 Dec 22;22:317. doi: 10.1186/s12931-021-01914-6 (PMC8692825; doi:10.1186/s12931-021-01914-6)
Supplement: Supplementary file 1 — Additional file 1: Figure S1. ΔWHO scale at day 10 was significantly lower in the tocilizumab group compared to the usual care group (− 0.5±2.1 vs 0.6±2.6, p=0.005), (A). ΔPaO2/FiO2 at day 5 was significantly higher in the tocilizumab group compared to the usual care group [42.0 (23.0–84.7) vs 15.8 (− 19.4–50.3), p=0.03], (B). Figure S2. Kaplan-Meier analysis in the overall population irrespective of treatment arm demonstrated higher all-cause mortality in patients with high (≥35) vs. low ΔPaO2/FiO2 at day 5 (< 5) [HR 3.70 (95% CI: 1.24–11.00), (p=0.03)]. [file 12931_2021_1914_MOESM1_ESM.docx]

**Tocilizumab improves 28-day survival in hospitalized patients with severe COVID-19: an open label, prospective study.**

Theodoros Karampitsakos^1*^, Elli Malakounidou^1*^, Ourania Papaioannou^1^, Vasilina Dimakopoulou^2^, Eirini Zarkadi^1^, Matthaios Katsaras^1^, Panagiota Tsiri^1^, Georgios Tsirikos^1^, Vasiliki Georgiopoulou^1^, Ioanna Oikonomou^2^, Christos Davoulos^2^, Dimitrios Velissaris^2^, Fotios Sampsonas^1^, Markos Marangos^2#^, Karolina Akinosoglou^2#^, Argyris Tzouvelekis^1#^

^1^ Department of Respiratory Medicine, University Hospital of Patras, Greece

^2^ Department of Internal Medicine, University Hospital of Patras, Greece

*equally contributed to this work

# jointly supervised this work

Correspondence to:

Argyrios Tzouvelekis MD, MSc, PhD

Associate Professor of Respiratory Medicine

Head Department of Respiratory Medicine

University of Patras, Greece

atzouvelekis@upatras.gr, argyrios.tzouvelekis@fleming.gr

**Additional file 1: Figure S1.** ΔWHO scale at day 10 was significantly lower in the tocilizumab group compared to the usual care group (-0.5±2.1 vs 0.6±2.6, p=0.005), **(Panel A)**. ΔPaO_2_/FiO_2_ at day 5 was significantly higher in the tocilizumab group compared to the usual care group [42.0 (23.0 to 84.7) vs 15.8 (-19.4 to 50.3), p=0.03], **(Panel B)**.

**Additional file 1: Figure S2.** Kaplan-Meier analysis in the overall population irrespective of treatment arm demonstrated higher all-cause mortality in patients with high (≥ 35) vs. low ΔPaO_2_/FiO_2_ at day 5 (< 35) [HR 3.70 (95% CI: 1.24 to 11.00), (p=0.03)].
